# Supplementary material for: Novel Agents as Main Drivers for Continued Improvement in Survival in Multiple Myeloma
Source: Cancers (Basel). 2023 Mar 2;15(5):1558. doi: 10.3390/cancers15051558 (PMC10000382; doi:10.3390/cancers15051558)
Supplement: Supplementary file 1 [file cancers-15-01558-s001.zip › cancers-2237655-supplementary.pdf]

Suplemmentary Table S1. Response after induction according to the treatment divided by age at diagnosis.

	**Age ≤70 years**		**Age >70 years**	
	≥PR after induction (%)	≥CR after induction (%)	≥PR after induction (%)	≥CR after induction (%)
**Conventional therapies**	80.3	20.1	68.0	16.8
**Novel agents**	92.8	39.6	87.1	34.7
**OR [95% CI], *P* value**	3.1 [1.9-5.2], <0.001	2.6 [1.8-3.7], <0.001	3.2 [1.7-6.1], <0.001	2.6 [1.4-4.8], 0.002
**Single novel agent inductions**	85.7	28.6	80.7	26.5
**At least two novel agent inductions**	96.9	46.0	100.0	51.2
**OR [95% CI], *P* value**	5.2 [2.1-12.8], <0.001	2.1 [1.3-3.4], 0.001	Not estimated, 0.001	2.9 [1.3-6.4], 0.007

Abbreviations: CI: confidence interval; CR: complete response; OR: odds ratio; PR: partial response.
